# Steps towards Preventive HIV Treatment in Fujian, China: Problems Identified via an Assessment of Initial Antiretroviral Therapy Provision

**DOI:** 10.1371/journal.pone.0076483

**Published:** 2013-09-24

**Authors:** Pincang Xia, Junko Okumura, Pingping Yan, Meirong Xie, Shouli Wu, Meizhu Zhuang, Jian Zheng, Chunyang Zhang, Mingya Zhang, Masaya Kato, GuoXi Cai, Taro Yamamoto

**Affiliations:** 1 Department of STDs & HIV/AIDS Control and Prevention, Fujian Center for Disease Control and Prevention, Fuzhou, Fujian Province, China; 2 Department of International Health, Institute of Tropical Medicine and Global Center of Excellence Program, Nagasaki University, Nagasaki, Japan; 3 Department of Disease Control and Prevention, Beitang District Center for Disease Control and Prevention, Wuxi, Jiangsu Province, China; 4 HIV Care and Treatment, World Health Organization Vietnam Country Office, Hanoi, Vietnam; Infectious Disease Service, United States of America

## Abstract

**Background:**

At the end of 2009, a total of 501 AIDS patients were receiving antiretroviral therapy (ART) in Fujian Province in China, yet there were no assessments to determine treatment efficacy and HIV-1 preventive potency under the current health care delivery system.

**Methods:**

During the period of 2005–2009, we assessed the outcomes of initial ART by following up 381 patients for 12 months in Fujian Province. CD4^+^ T-lymphocyte (CD4) count, plasma viral load (VL), and patient characteristics were analysed. The results were compared between 4 groups divided by the baseline CD4 values at the 25, 50 (median), and 75 percentiles.

**Findings:**

Over three-quarters of the subjects reported heterosexual contact as the probable route of transmission. After 12 months of ART, CD4 recovery varied between the 4 groups (*P* < 0.001), but VL sharply declined regardless of the baseline CD4 count (*P* = 0.136). Although this VL decline indicates the potency of ART as an HIV-1 prevention tool, the time between positive diagnosis and ART initiation suggests serious delay in both diagnosis and treatment; the medians of periods for the lowest and highest baseline CD4 quartiles were 1.2 and 9.6 months, respectively.

**Conclusion:**

Current limitations in VL determination make it difficult to assess the efficacy of initial ART, and delays in diagnosis and treatment suggest that subjects contributed to HIV-1 transmission while they were not receiving ART. The current National Free ART scheme does not provide free treatment for sexually transmitted infection (STI), and there is no link between ART and the STI care delivery system. This may interfere with the HIV-1 preventive potency of ART. We highly recommend establishing a collaborating mechanism with STI care, strengthening the VL determination system, and promoting HIV tests and early ART initiation.

## Introduction

At the end of 2011, the number of people living with HIV (PLHIV) in China was 780,000, accounting for 0.058% of the total population. With this low national prevalence rate, China has lower-level HIV endemics compared to many other countries, but the number of PLHIV and AIDS patients is continuously increasing. The major modes of transmission are sexual intercourse and injecting drug use. The proportions of PLHIV who contracted HIV-1 through sexual contact and injecting drug use are 75.2% and 17.3%, respectively [1]. Under these circumstances, an ongoing project entitled ‘HIV Testing and ART Treatment as Prevention Strategies’ was initiated in China in 2011 in order to assess the preventive effects of ART in sero-discordant couples [2]. To derive conclusions about efficacy, it is recommended that data over several years be analysed.

The epidemic level in Fujian Province is almost equivalent to the national level. However, according to Yao X et al., the number of HIV-1 infections in Fujian Province sharply increased from 528 in 2006–2007 to 1129 in 2008–2009 [3]. Although the proportion of injectable drug users among PLHIV decreased from 11.0% to 6.7% during this period, the proportion engaging in heterosexual contact increased from 51.5% to 66.0%. In Fujian province, the major mode of transmission is heterosexual intercourse [4,5]. The Fujian Center for Disease Control and Prevention actively used the available resources to stop transmission, such as conducting campaigns for raising public awareness by distributing educative materials with condoms; strengthening advocacy at workplaces, particularly, in the entertainment industry; promoting voluntary counselling and testing; promoting needle exchange programs and provision of methadone replacement therapy; and subsidising NGOs that distributed condoms [6]. Nevertheless, the number of PLHIV continues to rise. Reportedly, of the HIV-infected people in Fujian, the proportion of persons in whom HIV was transmitted by their HIV-positive spouse or (stable) partner was 17.3% in 2009 [3].

In this context, free ART was initiated in Fujian Province in 2005. At the end of 2009, a total of 501 AIDS patients were receiving therapy. However, there are no assessments of treatment efficacy or HIV-1 preventive potency under the current health care delivery system in Fujian Province. According to the national ART manual, the first 12 months of therapy is an important period for determining treatment efficacy [7]. Although several researchers reported the use of ART for HIV prevention [8-11], no study has examined it in the context of PLHIV in Fujian Province. Therefore, we conducted this study to assess the initial provision of ART in order to identify problems in the current system that interfere with the HIV-1 preventive potency of ART in Fujian Province. This is the first longitudinal study implemented to accomplish these objectives in Fujian Province.

## Methods

### Patients

**Figure 1:**
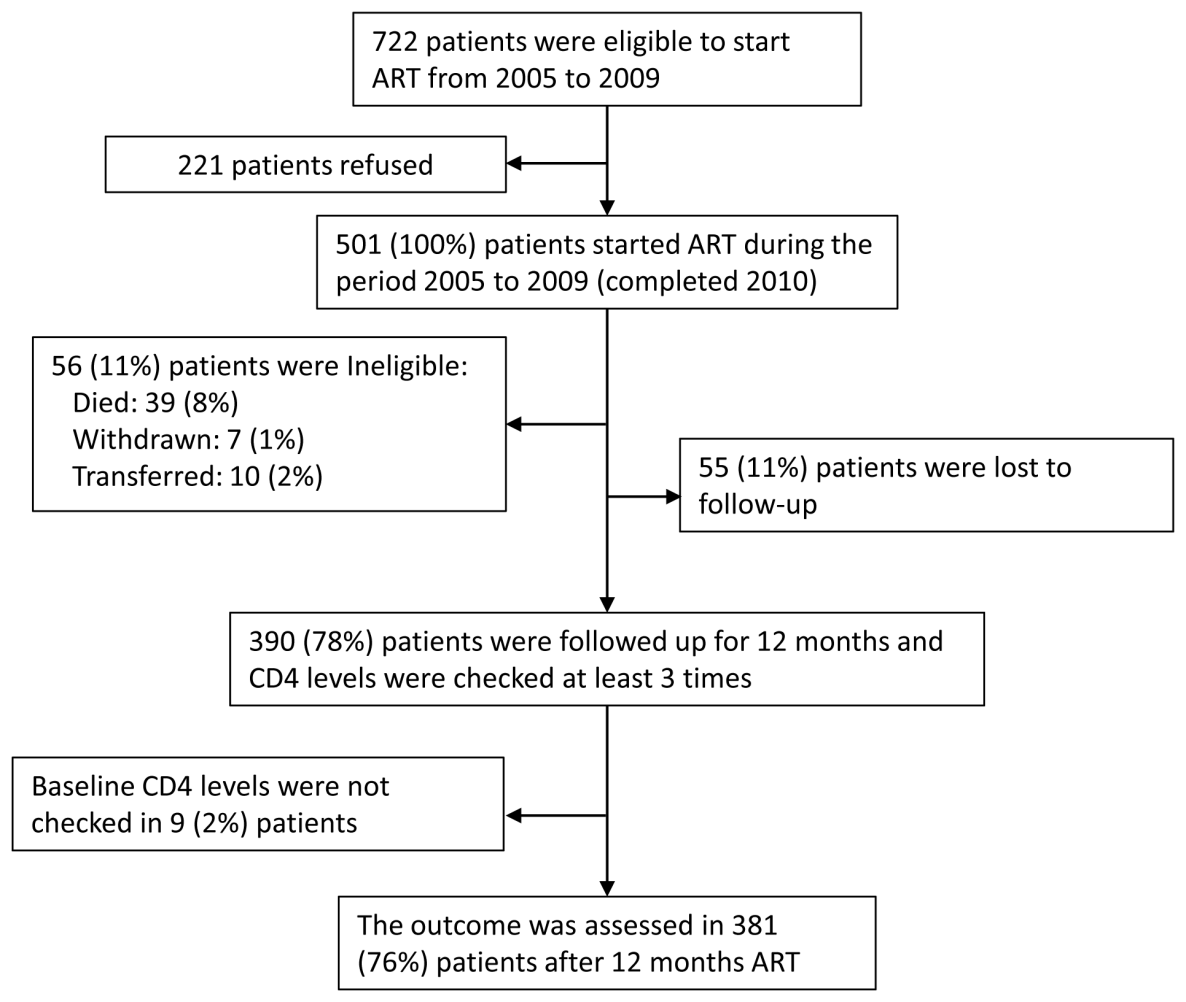
Flowchart of study subjects.

Patients who met the national treatment guidelines entry criteria were selected to initiate antiretroviral therapy (ART); these include a CD4^+^T-lymphocyte (CD4) count < 200/µl, a total lymphocyte count < 1200/µl, or HIV stage III clinical condition according to the World Health Organization (WHO) [7]. During the period, between February 2005 and December 2009, 722 patients were eligible to start ART. Of these, 221 refused to receive ART; therefore, 501 patients received ART. Each subject was evaluated prior to ART initiation and subsequently every 3 months up to 12 months. Of the 501, 56 (11%) were ineligible as they had less than 12 months of follow-up because of death (39 (8%)), withdrawal due to side effects (7 (1%)), or transfer to other provinces (10 (2%)). In addition, 55 (11%) patients were lost to follow-up. Nine subjects (2%) did not have their CD4 count measured before ART initiation and therefore had no baseline CD4 count. Altogether, 381 cases (76%) of 12-month ART were evaluated in this study (Figure 1). After 12 months of follow-up, all subjects continued on ART. In addition, all the injectable drug users were given methadone replacement therapy.

Four types of ART regimen were provided by the National Free Antiretroviral Therapy Program (NFATP). In this study, the regimen in ART-naïve patients was the first-line regimen that consists of 2 nucleoside reverse transcription inhibitors (NRTIs) and 1 non-nucleoside reverse transcription inhibitor (NNRTI) or 1 protease inhibitor (PI).

### Laboratory tests and interviews

At baseline and at each follow-up visit, all subjects were weighed and asked to provide blood specimens. These specimens were used to measure the CD4 count (BD TriTEST^TM^ CD3/CD4/CD45; Becton, Dickinson, USA) and viral load (VL) (Versant® HIV-1 RNA 3.0 Assay (bDNA); Siemens Healthcare Diagnostics Inc., USA) and for routine blood and liver function tests. Due to a logistical problem, during the follow-up period, plasma VL determination was obtained for only 126 subjects (33%).

Subjects were also interviewed at baseline. The main information collected were gender, age, probable route of infection, and date of HIV diagnosis as confirmed by western blotting (MP Diagnostics^TM^ HIV BLOT 2.2; MP Biomedicals, Asia Pacific Pte. Ltd., Singapore).

### Assessment and statistical analysis

The efficacy of the ART provided to patients was assessed using the WHO criteria for immunological and virological failure. The criteria for immunological failure are ‘fall of CD4 count to <100 cells/mm^3^,’ ‘50% fall of baseline CD4 from treatment peak value,’ or ‘fall to baseline CD4 level or below’. The criterion for virological failure is plasma viral load more than 3.7 log_10_ RNA copies/ml when a patient has received ART for at least 6 months [12]. Pearson’s chi-square test and the Kruskal-Wallis test were performed to assess differences in demographic data and changes in CD4 count and VL using the baseline CD4 count quartiles, which was divided by the baseline CD4 values at the 25, 50 (median), and 75 percentiles. All statistical analyses were performed using SPSS software, version 16.0 (SPSS Inc., Chicago, IL, USA). For all tests, *P* < 0.05 was considered to be statistically significant.

### Ethics Statement

The Health Research Ethics Committee of Fujian Center of Disease Control and Prevention approved the study and granted ethical approval in December 2004. All subjects were individually informed about the study procedures, and all patients signed informed consent forms. Confidentiality for the respondents’ information and blood test results was guaranteed.

## Results

### Characteristics of the subjects

There were 253 male (66%) and 128 female (34%) subjects, with a mean age of 39.0 ± 11.1 (SD). Of these, 67% (255/381) were married, 15% (57/381) had never married, 8% (31/381) were widowed, and 7% (27/381) were divorced. With regard to the probable route of transmission, 289 (76%) subjects stated that it was heterosexual intercourse, with 6% (21/381) attributing it to injecting drug use, 3% (11/381) to homosexual intercourse, and 1% (5/381) to blood transfusion. The baseline CD4 counts at the 25, 50 (median), and 75 percentiles were 30, 86, and 173 cells/mm^3^, respectively. Based on these values, the 381 subjects were divided into baseline CD4 count quartiles (Table 1).

**Table 1:** Subject characteristics.

**Category**	**n = 381**
**Gender**	
• Male	253 (66%)
• Female	128 (34%)
**Age (years**)**, mean ± SD**	39.0 ± 11.1
**Mode of transmission**	
• Blood transfusion	5 (1%)
• Injectable drug use	21 (6%)
• Homosexual	11 (3%)
• Heterosexual	289 (76%)
• Unknown	55 (14%)
**Marital status**	
• Never married	57 (15%)
• Married	255 (67%)
• Divorced	27 (7%)
• Widowed	31 (8%)
• Unknown	11 (3%)
**Baseline CD4 count (cells/mm^3^**)	
• 25 percentile value	30
• Median	86
• 75 percentile value	173
• Mean	118
• 95% Confidence Interval	106–130

SD: Standard deviation

### Comparison of ART outcomes

We compared ART outcomes between the 4 groups of baseline CD4 count quartiles. There were no differences in age (*P* = 0.776), gender (*P* = 0.841), or mode of transmission (*P* = 0.051) among the 4 groups. At baseline, the number of subjects reporting persistent diarrhoea did not differ among the 4 quartiles (*P* = 0.066), while the number with persistent fever significantly varied (*P* < 0.001) (Table 2). Those who had a clinical event of ‘stage 3’, based on WHO diagnostic criteria for HIV-related clinical events [12], after 12 months of ART were 6 patients with severe unexplained weight loss of more than 10%, and 1 patient with severe anaemia whose haemoglobin level was below 8 g/dl; and in whom neither neutropenia nor thrombocytopenia was observed (Figure S1).

**Table 2:** Characteristics of subjects by baseline CD4^+^ T-lymphocytes (CD4) levels (N=381).

	**Baseline CD4 count category(cells/mm^3^**)**^a^**				***P* value**
	**1st quartile**	**2nd quartile**	**3rd quartile**	**4th quartile**	
	**≤30**	**31–86**	**87–173**	**≥174**	
**Characteristics**	**n = 100**	**n = 91**	**n = 95**	**n = 95**	
**Age (years**)**, mean ± SD**	38.0 ± 10.8	39.9 ± 11.7	38.9 ± 11.0	39.0 ± 11.0	*P* = 0.776
**Gender**					
• Male	69 (69%)	62 (68%)	61 (64%)	61 (64%)	*P* = 0.841
• Female	31 (31%)	29 (32%)	34 (36%)	34 (36%)	
**Mode of transmission**					
• Blood transfusion	1 (1%)	4 (5%)	0 (0%)	0 (0%)	*P* = 0.051
• Injectable drug use	1 (1%)	7 (8%)	7 (7%)	6 (6%)	
• Homosexual	2 (2%)	2 (2%)	5 (5%)	2 (2%)	
• Heterosexual	79 (79%)	62 (67%)	70 (74%)	78 (82%)	
• Unknown	17 (17%)	16 (18%)	13 (14%)	9 (10%)	
**Time between western blot test positive and ART initiation (months**)**^b^**	1.2 (0.8–2.0)	1.6 (0.9–6.0)	6.1 (1.4–22.0)	9.6 (1.6–22.4)	*P* < 0.001
**Symptom at baseline**					
• Persistent diarrhoea	12 (12%)	14 (15%)	4 (4%)	8 (8%)	*P* = 0.066
• Persistent fever	50 (50%)	40 (44%)	23 (24%)	23 (24%)	*P* < 0.001

The figures indicate the number of cases, unless otherwise indicated.

a Based on the 25, 50 (median), and 75 percentile values of the baseline CD4 counts, the 381 subjects were divided into 4 groups.

b Median, (Interquartile range: 1^st^–3^rd^ Quartiles).

The baseline CD4 count varied significantly among the 4 groups (*P* < 0.001). The medians and interquartile ranges (1^st^–3^rd^ quartiles) for the first, second, third, and fourth quartile CD4 counts at baseline were 12.0 (7.0–19.8), 56.0 (43.0–73.0), 126.0 (110.0–149.0), and 220.0 (197.0–288.0) cells/mm^3^, respectively. The median CD4 counts at 12 months after ART initiation for the first, second, third, and fourth quartiles were 176.0 (123.0–230.0), 224.0 (141.0–294.0), 256.0 (202.0–335.0), and 352.5 (277.0–469.3) cells/mm^3^, respectively (*P* < 0.001). The number of patients whose CD4 count increment was more than 150 cells/mm^3^ after 12 months of ART varied significantly among the 4 groups (*P* = 0.001). Particularly, the proportion of the cases in the first quartile baseline CD4 group was almost twice that in the fourth quartile group (Table 3).

**Table 3:** ART outcome by baseline CD4 levels (N=381).

	**Baseline CD4 count category(cells/mm^3^**)				***P* value**
	**1st quartile**	**2nd quartile**	**3rd quartile**	**4th quartile**	
	**≤30**	**31–86**	**87–173**	**≥174**	
**Outcome**	**n = 100**	**n = 91**	**n = 95**	**n = 95**	
**CD4count(cells/mm^3^**)**^a^**					
• Baseline	12.0 (7.0–19.8)	56.0 (43.0–73.0)	126.0 (110.0–149.0)	220.0 (197.0–288.0)	*P* < 0.001
• After 12 months of ART	176.0 (123.0–230.0)	224.0 (141.0–294.0)	256.0 (202.0–335.0)	352.5 (277.0–469.3)	*P* < 0.001
**CD4 Increment ≥ 150 cells/mm** ^**3** b^	54 (54%)	41 (45%)	34 (36%)	26 (27%)	*P* = 0.001
**Immunological failure after 12 months of ART (%**)					
• CD4 count < 100 cells/mm^3^ (1)	16 (16%)	12 (13%)	5 (5%)	0 (0%)	*P* < 0.001
• 50% fall of CD4 level from treatment peak value (2)	2 (2%)	5 (5%)	4 (4%)	2 (2%)	*P* = 0.479
• Fall to baseline CD4 level or below (3)	0 (0%)	4 (4%)	6 (6%)	14 (15%)	*P*<0.001
• Immunological failure: either (1), (2), or (3)	16 (16%)	15 (16%)	9 (9%)	15 (16%)	*P* = 0.468
**Viral load (VL**)** (log_10_ RNA copies/ml**)**^a^**					
• Baseline ^c^	4.87 (4.00–5.82)	5.73 (5.04–5.94)	5.19 (4.95–6.20)	4.76 (4.02–5.34)	*P* = 0.029
	n = 27	n = 23	n = 14	n = 12	
• After ART was given^d^	1.70 (1.70–2.10)	2.12 (1.70–2.70)	2.05 (1.70–2.70)	1.70 (1.70–2.31)	*P* = 0.136
	n = 35	n = 28	n = 32	n = 31	

The figures indicate the number of cases, unless otherwise indicated.

a Median, (Interquartile range: 1^st^–3^rd^ Quartiles).

b According to the criteria developed by the Chinese CDC, it is expected that the CD4increment should be more than 150 cells/mm^3^ after 12 months of ART.

c Due to problems related to logistics and financial constraints, this test was not given to all subjects.

d After ART initiation, VL was measured only once or twice; the more recent value is indicated in the table. Cases with values less than the detection limits were included.

The median (interquartile ranges: 1^st^-3^rd^ quartiles) of VLs at the baseline for the first, second, third, and fourth quartile were 4.87 (4.00–5.82), 5.73 (5.04–5.94), 5.19 (4.95–6.20), and 4.76 (4.02–5.34) log_10_ RNA copies/mL, respectively. The levels sharply declined after ART initiation. After 12 months of ART, there were no differences in VL level among the 4 quartiles (*P* = 0.136). The median VLs at 12 months after ART initiation for the first, second, third, and fourth quartile were 1.70 (1.70–2.10), 2.12 (1.70–2.70), 2.05 (1.70–2.70), and 1.70 (1.70–2.31) log_10_ RNA copies/mL, respectively (Table 3).

### Assessment of ART outcome

The efficacy of ART was assessed in accordance with the WHO criteria for immunological failure [12]. There was no difference in the proportion that was judged as immunological failure among the 4 quartiles (*P* = 0.468), and the entire immunological failure rate was 14% (Table 3). Of the 126 subjects whose plasma VL determination was obtained, we assessed virological response and compared it with immunological response. The discordance rates for the first, second, third, and fourth quartiles of the baseline CD4 count category were 14%, 14%, 3%, and 13%, respectively (Table S1).

## Discussion

In summary, the mean age of the subjects was 39.0 years, 67% were married, and 76% of the self-reported probable route of transmission was heterosexual intercourse. After 12 months of ART, both the CD4 count and VL improved. While the CD4 increment depended on the baseline CD4 counts (*P* < 0.001), the VL decreased regardless of the baseline CD4 counts (*P* = 0.136). According to the Chinese criteria for the expected outcome of ART for naïve cases, the CD4 count increment should be more than 150 cells/mm^3^ after 12 months of ART [13]. The number of cases that met this criterion varied significantly among the baseline CD4 quartiles (*P* = 0.001). Some HIV clinical indicators, categorised as stage 3 according to WHO [12], such as chronic diarrhoea, persistent fever, neutropenia and thrombocytopenia were not observed, while severe weight loss (n = 6) and anaemia (n = 1) were observed 12 months after ART initiation. These facts reveal the efficacy of ART to a certain degree.

According to national and international authorities, more than 75% of PLHIV in China acquired the virus through heterosexual contacts [1]. This is also true for Fujian province, where HIV-1 infections are consistently related to unprotected sexual transmission [3]. Our results are consistent with these reports. As shown in Table 1, the proportion of PLHIV who acquired the virus through heterosexual contact was 76%, which is equivalent to the aforementioned national estimate. According to the national authorities, approximately 25% of those infected through heterosexual contact were infected by their spouse. According to the joint assessment conducted by the Ministry of Health, UNAIDS, and WHO, 32% of female sex workers reported that they and their clients did not use condoms consistently during transactional sex [1]. The self-reported route of transmission, i.e., mainly heterosexual intercourse, was consistent with the national situation. Based on the subjects’ condition with respect to chronic diarrhoea, persistent fever, neutropenia, thrombocytopenia, weight loss, and anaemia, majority of the subjects were not in a critical condition. These facts suggest that sexual intercourse with spouses should be a focus of further HIV transmission efforts in Fujian province in addition to commercial sex activities.

Several studies have indicated the effectiveness of ART provision in preventing HIV transmission to the general population [8-10,14]. The key point is that ‘The early initiation of antiretroviral therapy reduced rates of sexual transmission of HIV-1 and clinical events, indicating both personal and public health benefits from such therapy’ [9]. Even after the disclosure of serological status by HIV-positive partners, it has been reported that sero-discordant couples had sexual contact more than 3 times, and their condom use rate was not necessarily high [11]. Despite their poor immunological status, these people still have sexual relationships, frequently of an unprotected nature. Therefore, it is possible that our subjects might have had such relationships.

As Vernazza et al. stated, several conditions must be fulfilled in order to establish whether preventive ART is useful [10]; viraemia must be suppressed, i.e. ART should be effective, and HIV-positive individuals should be free from additional sexually transmitted infections (STIs). Since the current National Free ART scheme in China does not provide free treatment for other STIs, there were no data on STI diagnoses for the subjects in this study. Furthermore, plasma VL was only determined for less than one-third of the subjects in this study. Given these limitations, it is difficult to conclude with certainty that preventive ART is effective in Fujian. However, among a limited number of subjects whose VL was more than 3.70 log_10_ RNA copies/ml at the baseline, plasma VL reduced sharply and the virological failure rate was only 6% (7/126) after 12 months of ART. Regardless of the baseline CD4 count, the virological responses were almost of the same level (Table 3, Table S1). This virological response was consistent with that observed in previous studies [15-17]. Furthermore, the AIDS illness indicators were considered. After 12 months of ART, while persistent diarrhoea and fever observed at baseline diminished in all subjects (Table 2), 6 cases of severe weight loss, and 1 of severe anaemia were observed (Figure S1). Considering these, we cautiously conclude that except these 7 patients, the majority of the subjects responded to ART. Nevertheless, it was difficult to judge the efficacy of ART with the current limited data in this study. This limitation is a constraint in not just our analysis, but it is also a barrier in the assessment of ART in Fujian Province.

Plasma VL determination capability was limited due to the location where these tests are conducted. Tests should be initiated within 24 hours of the collection of plasma specimens, but specimens collected in health facilities located at a huge distance from the capital of Fujian could not be tested within this timeframe. To prevent further transmission, we recommend the establishment of linkage between free STI treatment and ART provision and expansion of the network of laboratories where plasma VL is measured.

Another strategy to control HIV transmission is the early detection of HIV status and early initiation of ART. The duration until ART initiation after a diagnosis of HIV was longer among the third and fourth CD4 count quartiles than among the first and second quartiles. Subjects in the third and fourth quartiles remained sources of HIV infection for more than 12 months. During this period, their plasma VL levels were high enough to transmit HIV (Table 3). This treatment delay may contribute to an increase in HIV transmission through sexual contact with spouses, partners, and commercial sex workers. To shorten this duration, the Chinese CDC recommends early ART initiation and has introduced the new ART enrolment criteria of a CD4 count below 350 cells/mm^3^ [13]. However, of the 772 patients in this study who were eligible to receive ART, 221 refused treatment (Figure 1). This refusal was caused by prevalent social stigma in Fujian and other provinces in China regarding HIV [18]. Furthermore, the first quartile appears to indicate diagnosis delay. When these subjects visited the clinic for sero-status tests, the majority had already developed AIDS; this diagnosis delay may have affected HIV incidence in Fujian. To enable early initiation of ART, more active advocacy appealing to both the personal and public health benefits from HIV tests and ART should be considered.

Although the CD4 count is an important factor in deciding whether to initiate ART in asymptomatic patients and assessing AIDS progression in PLHIV [16], accurate ART assessment cannot be conducted without data on plasma VL. As indicated in Table S1, there are clear gaps between immunological and virological failures. This gap makes it difficult to judge whether first-line ART should be changed to a second-line regimen [19,20].

In conclusion, we recommend establishing a collaboration with the free STI care system; expanding laboratory networks, particularly with regard to strengthening plasma VL determination; and providing more active public education in order to promote HIV tests and early ART initiation.

## Supporting Information

Figure S1 **Box plots of biological data by baseline CD4 count category.** Legend: ‘At Base’ means before ART initiation. ‘After 3 Months’, ‘After 6 Months’, ‘After 9 Months’ and ‘After 12 Months’ means after 3, 6, 9, and 12 months of ART.
(TIF)

Table S1 **Immunological failure and virological failure based on WHO criteria (N=112).** (DOCX)
