# Supplementary figures and images for: Steps towards Preventive HIV Treatment in Fujian, China: Problems Identified via an Assessment of Initial Antiretroviral Therapy Provision
Source: PLoS One. 2013 Sep 24;8(9):e76483. doi: 10.1371/journal.pone.0076483 (PMC3782456; doi:10.1371/journal.pone.0076483)

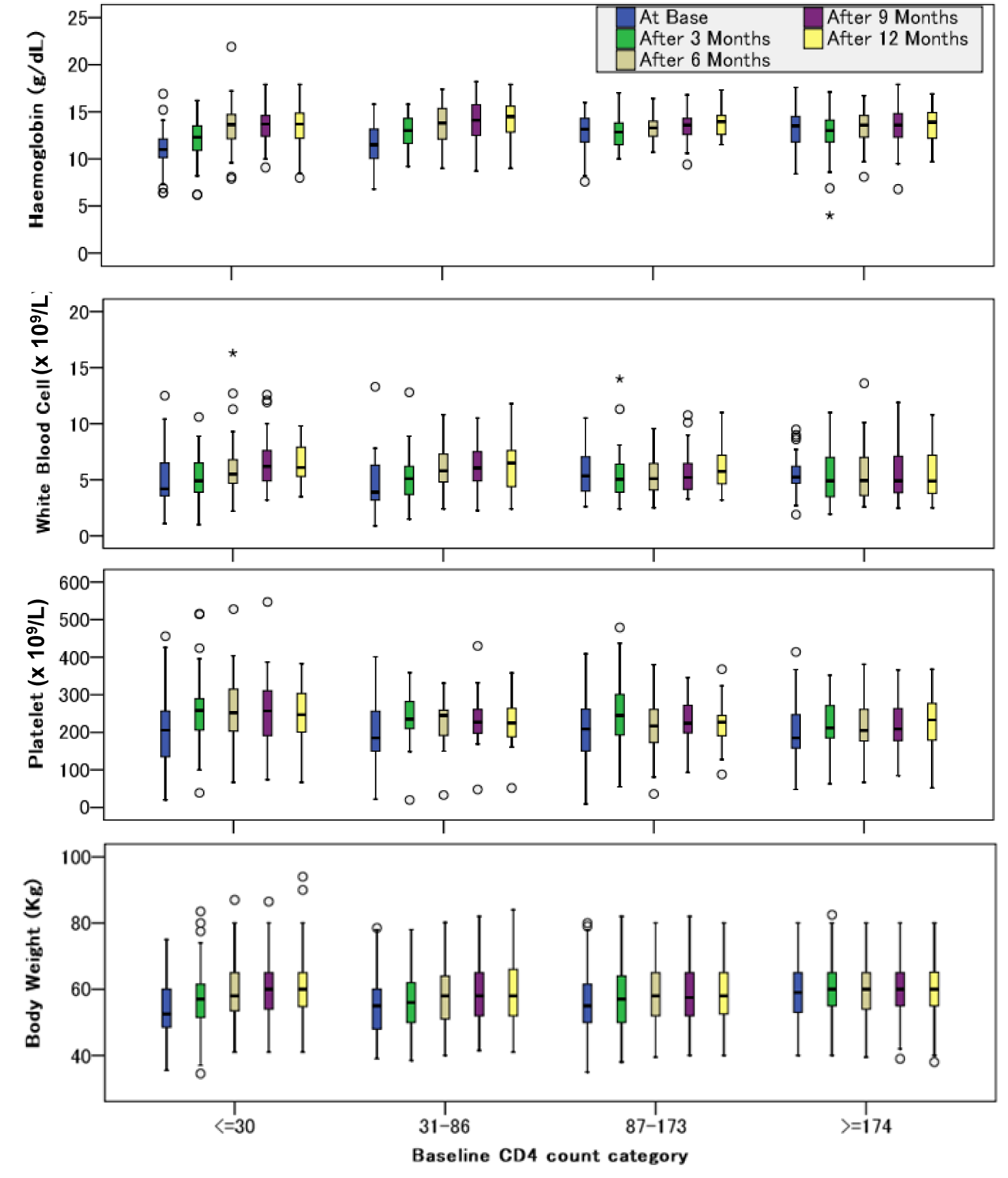

Supplement: Figure S1 — Box plots of biological data by baseline CD4 count category. Legend: ‘At Base’ means before ART initiation. ‘After 3 Months’, ‘After 6 Months’, ‘After 9 Months’ and ‘After 12 Months’ means after 3, 6, 9, and 12 months of ART. (TIF) [file pone.0076483.s001.tif]
