# Supplementary material for: Steps towards Preventive HIV Treatment in Fujian, China: Problems Identified via an Assessment of Initial Antiretroviral Therapy Provision
Source: PLoS One. 2013 Sep 24;8(9):e76483. doi: 10.1371/journal.pone.0076483 (PMC3782456; doi:10.1371/journal.pone.0076483)
Supplement: Table S1 — Immunological failure and virological failure based on WHO criteria (N=112). (DOCX) [file pone.0076483.s002.docx]

**Table S1. Immunological failure and virological failure based on WHO criteria (N = 112 ^a^)**

|  | Baseline CD4 count category (cells/mm^3^) | | | | *P*-value |
| --- | --- | --- | --- | --- | --- |
|  | 1st quartile  ≤30  n = 35 | 2nd quartile  31–86  n = 28 | 3rd quartile  87–173  n = 32 | 4th quartile  ≥174  n = 31 |  |
| Immunological failure (4) | 5 (14%) | 7 (25%) | 4 (13%) | 5 (16%) | *P* = 0.583 |
| Virological failure ^b^ (5) | 0 (0%) | 3 (11%) | 3 (9%) | 1 (3%) | *P* = 0.206 |
| Discordance between (4) and (5) | 5 (14%) | 4 (14%) | 1 (3%) | 4 (13%) | *P* = 0.431 |

^a^The number of subjects whose CD4 count and VL were measured was only 126.

^b^WHO criteria: When a patient has received ART for at least 6 months and plasma VL is more than 3.7 log_10_ RNA copies/ml, this is determined as ‘virological failure’
